# Antineoplastic Effects of Thymoquinone on Pancreatic Atypical Acinar Cell Foci Treated With Azaserine

**DOI:** 10.1002/jbt.70406

**Published:** 2025-07-23

**Authors:** Hasan Yildiz, Başak Hartavi

**Affiliations:** ^1^ Biology Department, Faculty of Arts and Sciences Hatay Mustafa Kemal University Hatay Turkey; ^2^ Graduate School of Natural and Applied Sciences Hatay Mustafa Kemal University Hatay Turkey

**Keywords:** AACF, antineoplastic effect, azaserine, exocrine pancreas, rat, thymoquinone

## Abstract

The antineoplastic effects of thymoquinone (TQ) (which has antioxidant and anticarcinogenic effects) on neoplastic changes (atypical acinar cell foci, AACF) induced by azaserine were investigated, for the first time. Rats were randomly divided into five groups of 10 rats each (Cont, Tim, Az, AzTim1, and AzTim2). The Cont group was fed only with a standard diet. TQ (50 mg/L) was given orally (P.O.) to the Tim, AzTim1, and AzTim2 groups. TQ was given to Tim and AzTim1 after the first month, while AzTim2 group rats were given after the third month of azaserine injection, when AACFs began to form. Azaserine was injected intraperitoneally (i.p.) (30 mg/kg bw) to 2‐weeks‐old Wistar albino male rats in the Az, AzTim1, and AzTim2 groups. AACF load were observed to be statistically significantly higher in all categories in the Az group compared to the Cont group (*p* < 0.05). AACF load were statistically significantly lower in all TQ groups (AzTim1 and AzTim2) compared to the Az group and it was demonstrated with TQ‐containing diet administration. This decrease indicates the antineoplastic effect of TQ on AACF and supports previous studies.

## Graphical abstract

The main purpose of this study was to investigate the antioxidant and anticarcinogenic effects of TQ, which has many traditional uses and is naturally found in *Nigella sativa* seeds, on the neoplastic changes induced by azaserine in exocrine pancreatic acinar cells. For this purpose, the anticarcinogenic effects of TQ given in the early period when AACFs were not yet formed (first month) and in the late period after AACFs were formed (third month) were investigated.

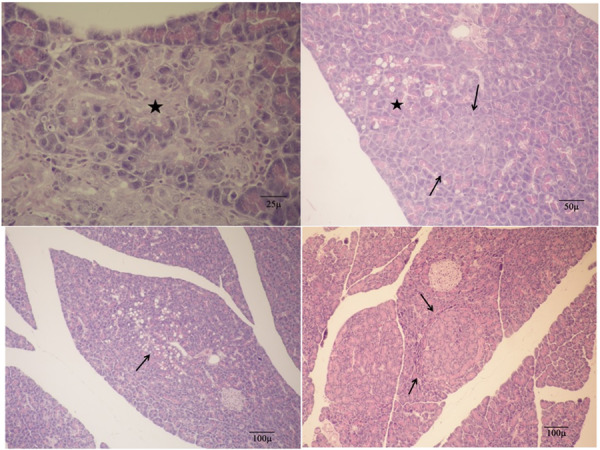

## 1 Introduction

Cancer is an important disease that threatens human wellness in our era. A lot of experimental, clinical, and epidemiological studies have been conducted for understanding the formation, progression and prevention of cancer. Previous studies have shown that thymoquinone (TQ), an active ingredient of the essential oil in *Nigella sativa* black seeds, may have antioxidant, anticarcinogenic, antimutagenic, and antimetastatic agent effects on neoplastic changes in human cells [1]. *Nigella sativa* L. (Ranunculaceae), known in Turkey and the Middle East, and black seeds are called black cumin and are often used as a seasoning agent or an alternative therapeutic agent. Preparations from black seed are widely used in the alternative treatment of many diseases in the Middle East and some Asian countries, as in our country [2]. Black seeds are widely used in alternative medicine, hypertension, rheumatism, burns, skin diseases, liver and kidney diseases, diabetes, gastrointestinal diseases, asthma, bronchitis, diarrhea, dyspepsia, headache, jaundice, and it has been applied to many diseases such as fever [3, 4].

Azaserine (o‐diazoacetyl‐l‐serine) is an antimetabolic substance obtained from Streptomycetes cultures and is known to have a mutagenic effect in Ames *Salmonella typhimurium* test [5]. Azaserine is assumed to exert its carcinogenic effects by inhibiting enzymes involved in DNA synthesis. Azaserin is an antimetabolite obtained from Streptomyces extracts with antibiotic properties and is effective as an inhibitor of purine ribonucleotide biosynthesis. A glutamine analog by structure, azaserine binds to enzymes involved in purine biosynthesis, competing with glutamine and inhibiting *de novo* purine biosynthesis [6].

The main purpose of this study was to investigate the antioxidant and anticarcinogenic effects of TQ, which has many traditional uses and is naturally found in *Nigella sativa* seeds, on the neoplastic changes induced by azaserine in exocrine pancreatic acinar cells. For this purpose, the anticarcinogenic effects of TQ given in the early period when atypical acinar cell focuses (AACFs) were not yet formed (first month) and in the late period after AACFs were formed (third month) were investigated.

## 2 Materials and Methods

### 2.1 Ethical Approval

Our study was conducted with the permission of Hatay Mustafa Kemal University Ethics Committee of Experimental Animals, dated 30/12/2015, No.2015/10‐12.

### 2.2 Animals

In this study, 2‐week‐old Wistar albino male rats were used to determine the antineoplastic changes thought to be caused by TQ in azaserine‐treated rats. Wistar albino male rats were bred in Hatay Mustafa Kemal University Experimental Research Application and Research Center (HMKU‐DAM).

### 2.3 Experimental Design

In this study, it was investigated how the development of AACF resulting from intraperitoneal injection of azaserine into 14‐day‐old male rats was affected by early (AzTim1) and late (AzTim2) diet restoration during a 5‐month experimental period. The control group rats (Cont) were fed with standard diet and 0.9% saline was injected intraperitoneally (i.p.). Rats in the TQ group (AzTim1, AzTim2) were fed TQ (P.O.) (50 mg/kg) after birth, in the early period (first month) when AACFs were not yet formed as a result of azaserine injection, and in the late period (third month) after AACFs were formed. In this way, the differences in the antineoplastic effect of TQ were investigated between the administration of TQ in the early period before AACFs were formed (AzTim1) and after AACFs had started to form (AzTim2). The rats used in the experiments were kept in cages in groups of five under standard conditions.

The rats used in the experiment were grouped as follows according to the purpose of the experiment:

**Cont:** Normal control group not injected with azaserine, fed with normal diet (*n* = 10);

**Tim:** Experimental group fed with TQ (P.O.) (50 mg/kg) from the first month (*n* = 10);

**Az:** Azaserine control group injected with azaserine (30 mg/kg bw) and fed with normal diet (*n* = 10);

**AzTim1:** Experimental group fed with TQ (P.O.) (50 mg/kg) from the first month and injected with azaserine (30 mg/kg bw) (*n* = 10);

**AzTim2:** Experimental group fed with TQ (P.O.) (50 mg/kg) from the third month and injected with azaserine (30 mg/kg bw) (*n* = 10).

### 2.4 Azaserine and Thymoquinone

Azaserine and thymoquinone were obtained from Sigma Aldrich Chemical Co. (St. Louis, MO; Cas 115‐02‐6 and 490‐91‐5).

### 2.5 Collection and Evaluation of Tissue Samples

At the end of the experiment (5 months), the rats were killed under ketamine–xylasine anesthesia. The pancreas was removed as a whole by abdominal dissection. The pancreas was fixed in 10% formalin for 24 h. After general tissue follow‐up, the tissues were blocked in soft paraffin and hematoxylin and eosin stain were applied to the sections taken from the prepared blocks with a thickness of 5 μm (Rotary microtome, Thermo Scientific, Shandon Finesse 325). Preparations were examined with Olympus brand BX‐51 research microscope and the Olympus C‐7070 camera was used to capture the necessary areas.

### 2.6 Statistical and Stereological Analysis

The Student‐Newman–Keuls Multiple Comparison Statistical Analysis (ANOVA) was used in the study (ProStat version 5.04 for Windows). The arithmetic mean ± standard deviation of the test results were given and *p* < 0.05 was considered as statistically significant. A mathematical formula was applied to determine atypical acinar cell focus characteristics (estimated mean diameter of foci [mm], estimated mean volume of foci [mm^3^], and estimated volume of foci as % of organ volume) in the pancreas. It is possible to calculate the approximate total tumor burden in the preparation based on the three‐dimensional structures of atypical acinar cell foci, tumors, and adenomas measured with the package program called VOLUGEN, modified by Campbell et al. and Pugh et al. [7, 8].

## 3 Results and Discussion

### 3.1 Quantitative Values of AACF

**FIGURE 1:**
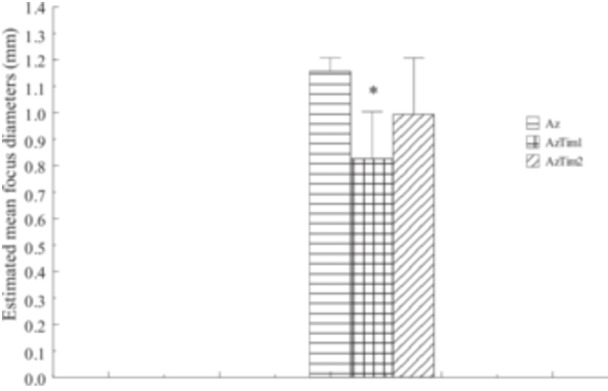
Estimated mean focus diameters of all groups (Az, AzTim1, and AzTim2) (mm). *A group that is statistically different from the Az group. No focus was found in the rat pancreas in the Con group and Tim group.

**FIGURE 2:**
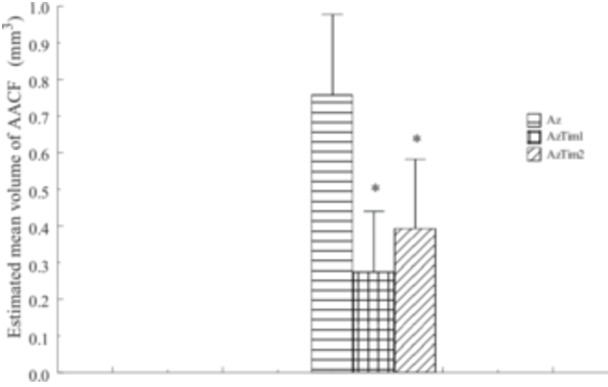
Estimated mean volume of AACF (mm^3^) of all groups (Az, AzTim1, and AzTim2). *A group that is statistically different from the Az group. No focus was found in the rat pancreas in the Cont group and Tim group.

**FIGURE 3:**
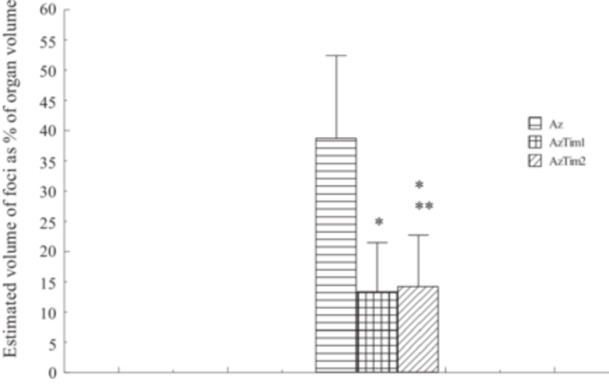
Estimated volume of foci as % of organ volume (Az, AzTim1, and AzTim2). *A group that is statistically different from the Az group. **Statistically not different from the Cont group. No focus was found in the rat pancreas in the Cont group and Tim group.

The values of estimated mean diameter (mm), estimated mean volume of foci (mm^3^) and estimated volume of foci as % of organ volume are shown in Figures 1, 2, 3, respectively. Quantitative values of AACF obtained from all groups were compared with those obtained from Az Group rats. Accordingly, as a result of TQ administration, a decrease in all values shown was observed in the AzTim1 group rats compared to the Az group. The difference between AzTim1 and Az was statistically significant (*p* < 0.05). Similarly, a decrease in all values was observed when the AzTim2 group values were compared with those of the Az group. The decrease in these values was statistically significant except for the mean focus diameters. According to these statistical data, both early and late TQ intake has an effect on the values of AACFs such as area and volume. However, early TQ intake is more effective on AACF than late TQ intake.

### 3.2 Histopathology of Atypical Acinar Cell Foci

As expected, no AACF was found in the pancreases of the Cont and Tim group rats as a result of the examinations. The difference in tumor burden between the Cont and Az group rats were statistically significant in all categories (*p* < 0.05). According to this, as a result of the histopathological examinations, it was observed that AACFs occurred in all azaserine groups (Az, AzTim1, and AzTim2).

**FIGURE 4:**
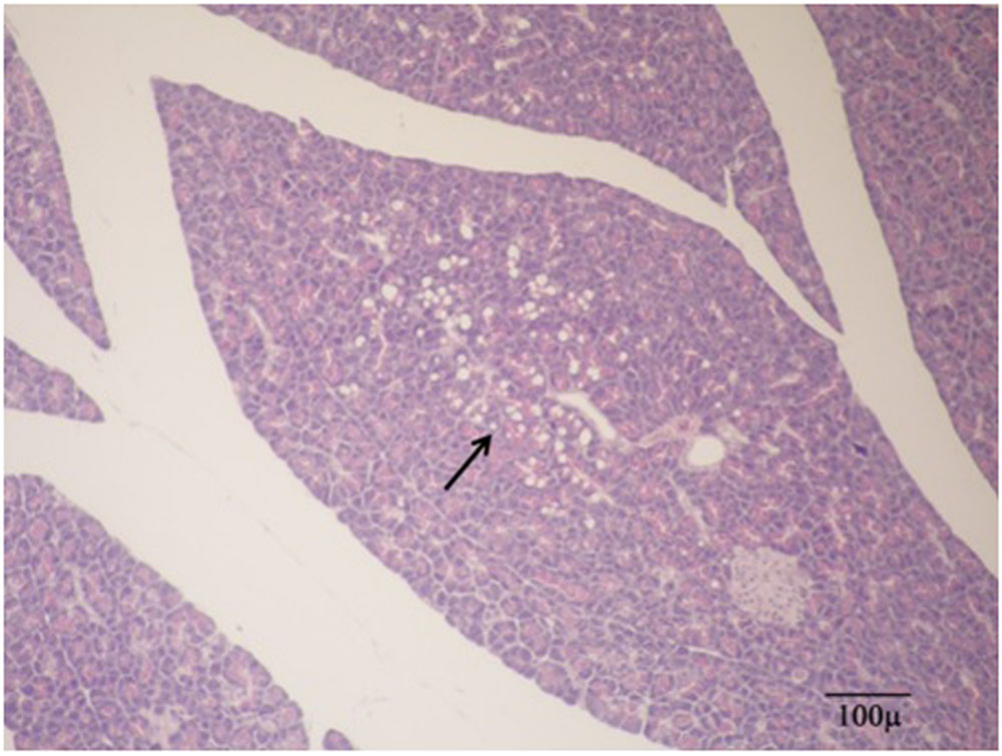
Abnormal tissue losses belonging to the AzTim2 group rat and easily distinguishable from the normal acinar tissue around it (H&E).

**FIGURE 5:**
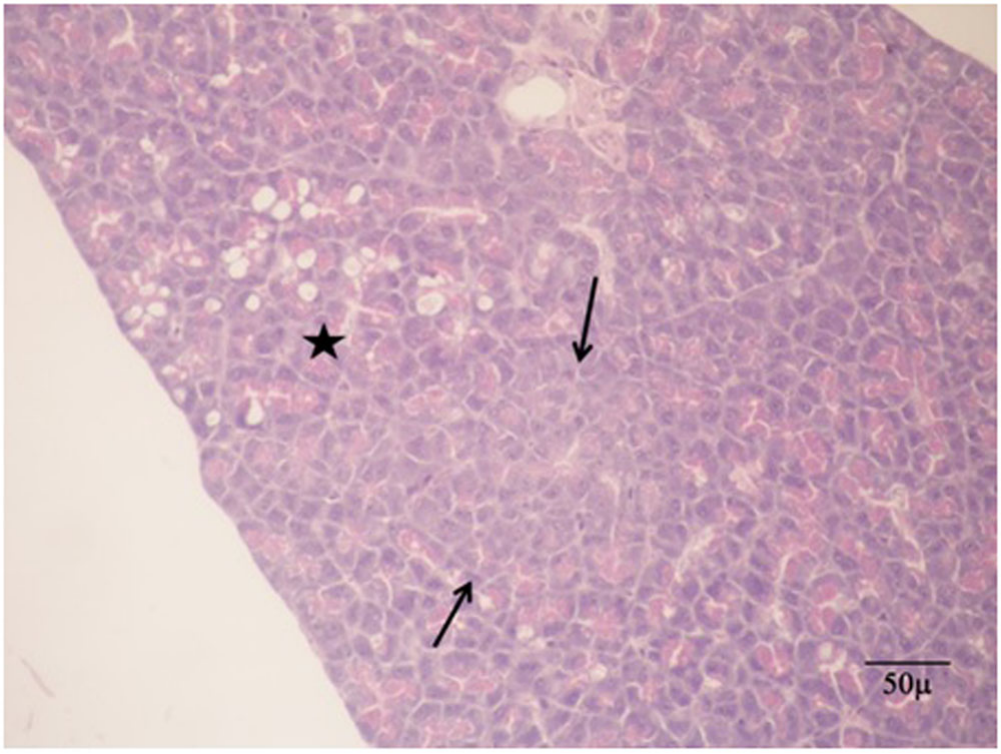
AzTim2 group of rats with diminishing zymogen granule number and pale appearance of the surrounding normal acinar cells different from the acinar cell focus (arrows) and the resulting loss of tissue caused by the effect of azaserine (Star) (H&E).

**FIGURE 6:**
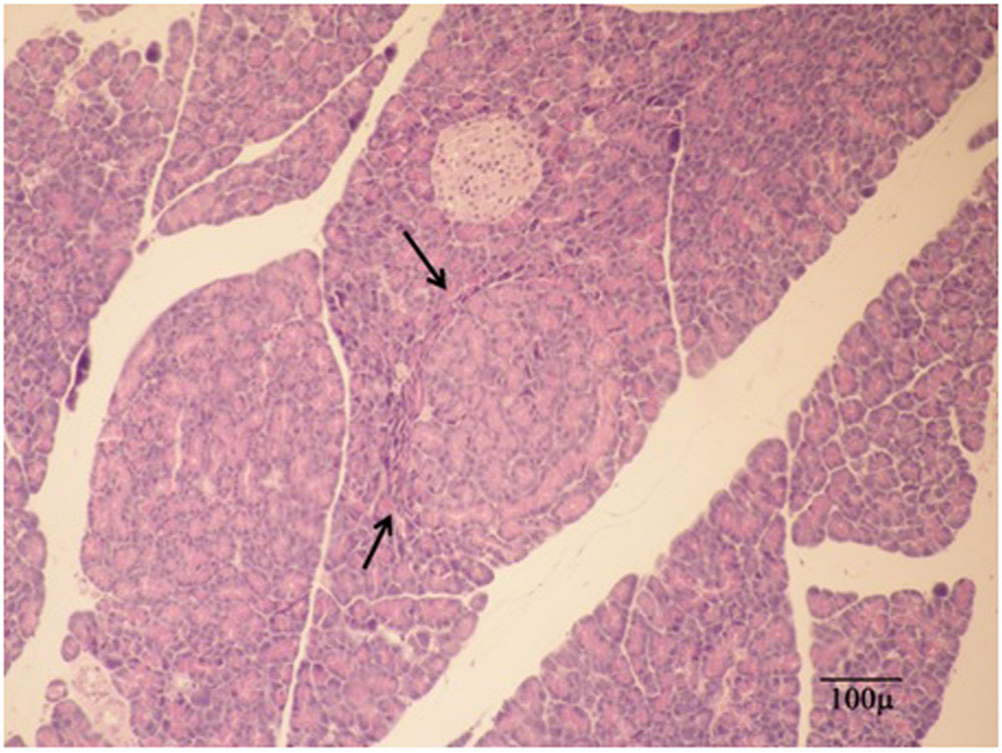
Atypic acinar cell focus (AACF) of the AzTim1 group of rats with a partially circular capsule‐free appearance is observed, slightly compressing the surrounding normal parenchyma upwards (arrows) (H&E).

**FIGURE 7:**
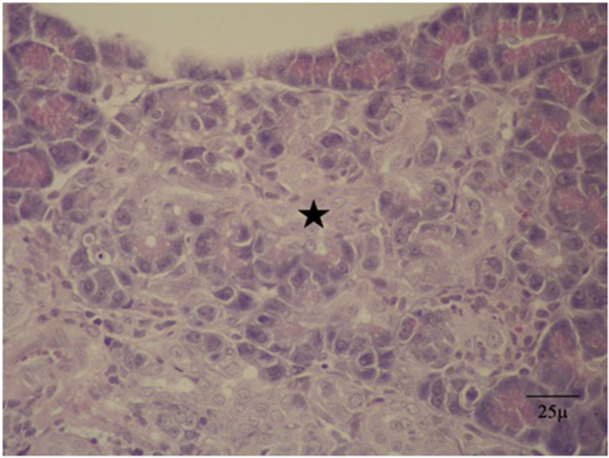
AzTim2 group of rats with diminishing zymogen granules and pale appearance and normal acinar cells around them that gained a different appearance feature focus (AACF) structure (Star) (H&E).

Disruption of the acinar parenchymal tissue was observed in the AzTim2 group rats (Figures 4 and 5). It was seen that the cells shrank in volume and there was a decrease in the core volumes. AACF with a partially circular capsule‐free appearance in the AzTim1 group rat pancreas appeared to slightly compress the normal parenchyma around it (Figure 6). In the Az Group rats (Az), it was observed that the foci characterized by a larger volume and basophil turn into relatively smaller foci in the AzTim1 and AzTim2 groups (Figure 7).

The number of new cancer cases worldwide was estimated as 8.1 million in 2018. Pancreatic cancer is the seventh most deadly cancer worldwide and patients of pancreatic cancer has very high mortality rate (an overall 5‐year survival rate of 9%). Pancreatic cancer has a striking statistic compared to all cancers. In 2018, approximately 459,000 cases of pancreatic ductal adenocarcinoma were diagnosed worldwide, and when the incidence rates were examined, it was found to be four times higher in highly developed countries than in less developed countries in both genders [9].

As with most other types of cancer, the most important causes of pancreatic cancer are DNA damage caused by inheritance, smoking or mutations caused by other lifestyle factors. Since the DNA damage is induced by oxidants in the metabolic process, the inhibitory effects of antioxidant nutrients on DNA damage and carcinogenesis have been tried to be demonstrated in a wide range of studies. Experimental studies have shown that antioxidant nutrients acting as anticarcinogenic agents can reduce the effect of oxidative agents leading to DNA damage, and these antioxidants can prevent or reduce cancer development [9].

Apoptosis is programmed cell death and occurs in multicellular organisms. Cells die by being altered as a result of some biochemical events. Cellular apoptosis occurs due to cell receptors and intracellular signals. Some researchers suggested that thymoquinone enhances natural killer cell activities and that thymoquinone exhibits high toxicity to the WEHI‐3 cell line, known for its early apoptosis increase [10]. In the same study, Bcl2 upregulation of the antiapoptotic protein and Bax downregulation of the apoptotic protein were observed with the effect of thymoquinone, and findings from the histopathology of the spleen and liver showed that thymoquinone inhibited WEHI‐3 growth in BALB/c mice. In parallel with the results of our study, it is possible to say that thymoquinone, which is thought to have an anticarcinogenic effect, can reduce neoplastic development in different tissues by causing apoptosis. Similarly, Rachel and Ashley (2014) showed that thymoquinone induced apoptosis and anticancer activity in animal and cellular models. They identified the effects of thymoquinone, which shares the structural properties of the main bioactive compound of black seed with 1,4‐benzoquinone and covalent topoisomerase II poisons, on human topoisomerase IIIa, as well as some other anticancer drugs targeting type II topoisomerases [11].

In another study examining the effect of thymoquinone on chemotherapy drugs, it was found that thymoquinone significantly increased the effects of Gemcitabine and Oxaliplatin drugs used in the treatment of pancreas cancer and this increase effect caused cell apoptosis [12].

Researchers demonstrated that the anti‐inflammatory and anticancer effects of thymoquinone increased the expression of many genes for NF‐regulation and regulation of the angiogenic (vascular endothelial growth factor) pathway. Thymoquinone recognized that its mechanism of action influenced its anticancer effects by inhibiting this NF‐κβ activation [13].

In an anticancer study on human cytomegalovirus (HCMV) with a hybrid of a number of active substances including CEM/ADR5000 (human leukemia cells) and thymoquinone, all hybrids showed very good activity in the submicromolar to micromolar range. Hybridization of thymoquinone, a natural product with anticancer activity, has been reported as a therapeutic agent that may have important contributions in the development of anticancer agents [14].

Studies have shown that thymoquinone can inhibit the growth of several cancer types such as pancreatic, breast, colon, prostate, lung, and hematological malignancies [15, 16].

The azaserine‐rat model is well known and has been used many times by researchers in similar studies. It has made significant contributions to revealing the properties of a therapeutic agent, whose antineoplastic effect on pancreatic acinar cell‐derived lesions has been investigated. Studies conducted on this model may provide early detection of benign or malignant potential of these changes by examining neoplastic changes and taking preventive measures [17, 18, 19].

## 4 Conclusions

According to the information obtained in previous studies, it is possible to say that thymoquinone induces apoptosis and anticancer activity, increases natural killer cell activities, stimulates the upregulation of the antiapoptotic protein Bcl2 and the downregulation of the apoptotic protein Bax, and thus can reduce the number, diameter or volume of pancreatic foci, as in many other tissues. In rats given azaserine, TQ intake (especially in the early weeks) supports that it can reduce the volume and diameter of the foci. As a result of our study, we see that long‐term use of TQ reduces the number, area, and volume of AACF in the exocrine pancreas of azaserin‐treated rats. Our findings indicated that TQ could inhibit (or at least help inhibit) exocrine neoplastic development in the rat exocrine pancreas. The results obtained confirm our prediction that TQ, especially its use from the early stages, can reduce tumor burden in the pancreas. To date, no detailed study has been conducted with the azaserine‐rat experimental model used in pancreatic cancer research.

Therefore, it is suggested that it would be useful to further investigate the possible neoplastic development inhibitory effects of TQ in the exocrine pancreas with the help of this well‐known experimental animal model.

## Author Contributions

Hasan Yıldız and Başak Hartavi performed all the experiments and drafted the main manuscript text. Hasan Yıldız constructed final versions of images and graphs and designed the experimental work, reviewed and approved the final version of the manuscript. Başak Hartavi calculated Quantitative Values of AACF, reviewed and approved the final version of the manuscript.

## Conflicts of Interest

The authors declare no conflicts of interest.

## Data Availability

The data that support the findings of this study are available from the corresponding author upon reasonable request.

## References

[1] B. H. Ali and G. Blunden , “Pharmacological and Toxicological Properties of *Nigella sativa* ,” Phytotherapy Research 17, no. 4 (2003): 299–305, 10.1002/ptr.1309. 12722128

[2] A. Abedi , M. Rismanchi , M. Shahdoostkhany , A. Mohammadi , and A. M. Mortazavian , “Microwave‐Assisted Extraction of *Nigella sativa* L. Essential Oil and Evaluation of Its Antioxidant Activity,” Journal of Food Science and Technology 54 (2003): 3779–3790, 10.1007/s13197-017-2718-1. PMC5643791 29085120

[3] M. F. Ramadan , “Nutritional Value, Functional Properties and Nutraceutical Applications of Black Cumin (*Nigella sativa* L.): An Overview,” International Journal of Food Science and Technology 42, no. 10 (2007): 1208–1218, 10.1111/j.1365-2621.2006.01417.x.

[4] R. Schneider‐Stock , I. H. Fakhoury , A. M. Zaki , C. O. El‐Baba , and H. U. Gali‐Muhtasib , “Thymoquinone: Fifty Years of Success in the Battle Against Cancer Models,” Drug Discovery Today 19 (2014): 18–30, 10.1016/j.drudis.2013.08.021. 24001594

[5] D. S. Longnecker and T. J. Curphey , “Adenocarcinoma of the Pancreas in Azaserine‐Treated Rats,” Cancer Research 35 (1975): 2249–2258. 1097106

[6] A. Kornberg and T. W. H. Baker , DNA Replication, 2nd ed. (Freemanand Co., 1992).

[7] H. A. Campbell , H. C. Pitot , V. R. Potter , and B. A. Laishes , “Application of Quantitative Stereology to the Evaluation of Enzyme‐Altered Foci in Rat Liver,” Cancer Research 42 (1982): 465–472. 6120037

[8] T. D. Pugh , J. H. King , H. Koen , et al., “Reliable Stereological Method for Estimating the Number of Microscopic Hepatocellular Foci From Their Transections,” Cancer Research 43 (1983): 1261–1268. 6825098

[9] C. P. Wild , E. Weiderpass , and B. W. Stewart , eds. World Cancer Report (WCR): Cancer Research for Cancer Prevention (International Agency for Research on Cancer, 2020). 39432694

[10] L. Z. Salim , R. Othman , M. A. Abdulla , et al., “Thymoquinone Inhibits Murine Leukemia WEHI‐3 Cells In Vivo and In Vitro,” PLoS One 10, no. 3 (2014): 10.1371/journal.pone.0115340. PMC4274020 25531768

[11] R. E. Ashley and N. Osheroff , “Natural Products as Topoisomerase II Poisons: Effects of Thymoquinone on DNA Cleavage Mediated by Human Topoisomerase IIα,” Chemical Research in Toxicology 27, no. 5 (2014): 787–793, 10.1021/tx400453v. 24650156 PMC4033629

[12] S. Banerjee , A. O. Kaseb , Z. Wang , et al., “Antitumor Activity of Gemcitabine and Oxaliplatin Is Augmented by Thymoquinone in Pancreatic Cancer,” Cancer Research 69 (2009): 5575–5583, 10.1158/0008-5472.CAN-08-4235. 19549912

[13] G. Sethi , K. S. Ahn , and B. B. Aggarwal , “Targeting Nuclear Factor‐κB Activation Pathway by Thymoquinone: Role in Suppression of Antiapoptotic Gene Products and Enhancement of Apoptosis,” Molecular Cancer Research 6 (2008): 1059–1070, 10.1158/1541-7786.MCR-07-2088. 18567808

[14] A. Ç. Karagöz , C. Reiter , E. J. Seo , et al., “Access to New Highly Potent Antileukemia, Antiviral and Antimalarial Agents via Hybridization of Natural Products (Homo)egonol, Thymoquinone and Artemisinin,” Bioorganic & Medicinal Chemistry 26 (2020): 3610–3618, 10.1016/J.BMC.2018.05.041. 29887512

[15] M. K. Shanmugam , J. H. Lee , E. Z. P. Chai , et al., “Cancer Prevention and Therapy Through the Modulation of Transcription Factors by Bioactive Natural Compounds,” Seminars in Cancer Biology 40–41 (2016): 35–47, 10.1016/j.semcancer.2016.03.005. 27038646

[16] M. K. Shanmugam , F. Arfusob , A. P. Kumara , et al., “Modulation of Diverse Oncogenic Transcription Factors by Thymoquinone, an Essential Oil Compound Isolated From the Seeds of *Nigella sativa* Linn,” Pharmacological Research 129 (2018): 357–364, 10.1016/j.phrs.2017.11.023. 29162539

[17] H. Yıldız , H. Oztas , D. Yıldız , A. Koc , and E. Kalipci , “Inhibitory Effects of Acetylsalicylic Acid on Exocrine Pancreatic Carcinogenesis,” Biotechnic & Histochemistry: Official Publication of the Biological Stain Commission 88 (2013): 132–137. 23331184 10.3109/10520295.2012.758779

[18] M. N. U. Coral , S. Ucman , Y. Hasan , O. Haydar , and D. Semih , “Potential Neoplastic Effects of Parathion‐Methyl on Rat Liver,” Journal of Environmental Sciences 21 (2009): 696–699. 10.1016/s1001-0742(08)62326-8 20108674

[19] Y. Yener , E. Kalıpcı , H. Öztaş , A. Aydın , and H. Yıldız , “Possible Neoplastic Effects of Acrylamide on Rat Exocrine Pancreas,” Biotechnic & Histochemistry 88, no. 1 (2012): 47–53, 10.3109/10520295.2012.733028. 23101568

